# Risk Stratification for Rebleeding in Obscure Gastrointestinal Bleeding After Comprehensive Gastrointestinal Evaluation

**DOI:** 10.1002/deo2.70378

**Published:** 2026-07-06

**Authors:** Sachiyo Onishi, Kiichi Otani, Naoya Masuda, Hiroki Taniguchi, Kentaro Kojima, Jun Takada, Masaya Kubota, Takashi Ibuka, Masahito Shimizu

**Affiliations:** ^1^ First Department of Internal Medicine Gifu University Hospital Gifu Japan

**Keywords:** capsule endoscopy, obscure gastrointestinal bleeding, rebleeding, risk scoring system, small‐bowel endoscopy

## Abstract

**Background and Aim**: Obscure gastrointestinal bleeding (OGIB) is defined as bleeding of unknown origin after comprehensive upper, lower, and small‐bowel evaluations. However, the incidence and predictors of rebleeding after negative comprehensive gastrointestinal evaluation remain unclear. We aimed to identify predictors of rebleeding and develop a simple risk stratification score for OGIB.

**Methods**: We retrospectively analyzed 226 patients diagnosed with OGIB between 2009 and 2024. The primary outcome was rebleeding during follow‐up. Independent predictors were identified using multivariable Cox proportional hazards analysis. A risk score was constructed from regression coefficients and evaluated using receiver operating characteristic analysis and Kaplan–Meier curves.

**Results**: Among 226 patients, 87 (38.5%) had overt OGIB and 139 (61.5%) had occult OGIB. Rebleeding occurred in 30 (13.2%) patients. Multivariable analysis identified liver cirrhosis (*β* = 0.59, *p* < 0.01), overt OGIB (*β* = 0.69, *p* < 0.01), and aspirin use (*β* = 0.51, *p* = 0.02) as independent predictors. Each factor was assigned one point, yielding a 0–3 point score. The score showed modest discriminative ability (area under the curve = 0.68). Rebleeding rates increased progressively with increasing scores (5.6%, 17.5%, 27.3%, and 100% for scores 0, 1, 2, and 3, respectively) and were significantly higher in the high‐risk group (*p* < 0.01).

**Conclusion**: Rebleeding remained clinically relevant despite comprehensive gastrointestinal evaluation. The proposed risk score provides a simple and practical tool for identifying patients at increased risk of rebleeding and may support risk‐adapted follow‐up after a negative comprehensive gastrointestinal evaluation.

**Trial Registration**: N/A.

**Abbreviations**
BAE: balloon‐assisted endoscopy
CE: capsule endoscopy
CS: colonoscopy
EGD: esophagogastroduodenoscopy
*GIFU*
: rebleeding risk stratification score
OGIB: obscure gastrointestinal bleeding
ROC: receiver operating characteristic

## 1 Introduction

Obscure gastrointestinal bleeding (OGIB) presents considerable diagnostic and therapeutic challenges in clinical practice. Traditionally, OGIB has been defined as gastrointestinal bleeding of unknown origin that persists or recurs after negative findings on conventional esophagogastroduodenoscopy (EGD) and colonoscopy (CS) [1, 2]. With recent advances in small‐bowel evaluation, including capsule endoscopy (CE) and balloon‐assisted endoscopy (BAE), visualization of the entire small intestine has markedly improved. Nevertheless, rebleeding continues to occur in a subset of patients even after a comprehensive evaluation of the entire gastrointestinal tract and failure to identify the source of bleeding.

In response to these diagnostic advances, the definition of OGIB in Japan has been revised to include cases in which no bleeding source is identified despite complete evaluation of the upper, lower, and small bowels [3]. This revision reflects the increasing importance of comprehensive small‐bowel evaluation in the diagnosis of OGIB. However, many previous studies evaluating rebleeding and its associated risk factors were conducted using earlier definitions, in which small‐bowel evaluation was often incomplete. Consequently, clinical characteristics and predictors of rebleeding in patients undergoing comprehensive gastrointestinal evaluations remain insufficiently understood.

Rebleeding is an important determinant of long‐term outcomes in OGIB and may result in anemia, repeated hospitalizations, and increased healthcare burden. Reported rebleeding rates vary considerably across studies, likely due to differences in patient populations, diagnostic strategies, treatment approaches, and definitions of OGIB [4, 5, 6, 7]. Several factors, including vascular lesions, comorbidities such as liver or cardiovascular disease [8, 9], and the use of antithrombotic agents [10], have been associated with rebleeding. However, robust evidence regarding the predictors of rebleeding, specifically in patients with negative findings after comprehensive gastrointestinal evaluation, remains limited. In addition, practical risk stratification tools applicable to routine clinical practice are lacking.

Considering the clinical impact of rebleeding and the need for individualized follow‐up strategies, the development of a simple and reliable risk‐stratification model is clinically important. Therefore, this study aimed to determine the incidence of rebleeding in patients with OGIB diagnosed using the revised Japanese definition, to identify independent predictors of rebleeding using multivariate Cox proportional hazards analysis, and to develop a practical risk‐stratification score for clinical use.

## 2 Methods

### 2.1 Study Design and Ethics Statement

This retrospective observational study was conducted at the Gifu University Hospital. The study protocol was reviewed and approved by the Institutional Review Board of Gifu University Hospital and was performed in accordance with the ethical principles of the Declaration of Helsinki [11]. Given the retrospective nature of the study, the requirement for written informed consent was waived, and informed consent was obtained using an opt‐out approach.

### 2.2 Study Population

We retrospectively reviewed consecutive patients who were suspected of having OGIB and underwent small‐bowel evaluation with CE and/or BAE at our institution between 2009 and 2024.

**FIGURE 1:**
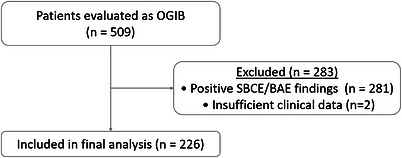
Flow diagram of patient selection. Among 509 patients initially evaluated as obscure gastrointestinal bleeding (OGIB) according to the former definition, 281 patients were excluded because a bleeding source was identified during capsule endoscopy (CE) and/or balloon‐assisted enteroscopy (BAE), and two patients were excluded because of insufficient clinical data. The remaining 226 patients were included in the final analysis.

During the study period, 509 patients fulfilled the conventional definition of OGIB, which was based on negative EGD and CS findings. Patients in whom a bleeding source was identified during CE and/or BAE evaluation were excluded. In addition, patients with insufficient clinical data were excluded. Ultimately, 226 patients met the inclusion criteria and were included in the analysis (Figure 1).

### 2.3 Endoscopic Evaluation

All patients underwent a comprehensive endoscopic evaluation of the entire gastrointestinal tract, including EGD, CS, and small‐bowel examination. Small‐bowel examinations were performed using CE and/or BAE according to clinical presentation and institutional practice.

CE was performed using the PillCam system (Medtronic, Dublin, Ireland). BAE was performed using a therapeutic double‐balloon system (Fujifilm, Tokyo, Japan).

CE findings were assessed using the modified Saurin classification, as previously reported by our group [12]. Negative findings were defined as the absence of any lesions considered to represent a definitive bleeding source according to the modified Saurin P0–P2 categories. Two experienced gastroenterologists independently reviewed all the CE images. In cases in which the interpretation was inconclusive or discordant, the final diagnosis was determined through discussion among experienced gastroenterologists.

BAE was performed selectively based on clinical indications to further evaluate the small bowel. However, in the patients included in this study, no definitive bleeding source was identified at the initial comprehensive evaluation.

### 2.4 Definitions and Outcome

Rebleeding was classified as overt or occult, according to standard definitions [13]. Overt OGIB was defined as clinically evident gastrointestinal bleeding, such as hematemesis or melena, whereas occult OGIB was defined as a decrease in hemoglobin level of ≥ 2.0 g/dL from baseline, unexplained by other causes, such as hematologic disease or acute illness.

The primary outcome was rebleeding during the follow‐up period. The time to rebleeding was calculated from the date of completion of the initial comprehensive evaluation to the first documented rebleeding event or last clinical follow‐up. Follow‐up data were obtained from electronic medical records and outpatient follow‐up visits.

### 2.5 Statistical Analysis

Continuous variables are expressed as medians with interquartile ranges, and categorical variables are presented as numbers and percentages. Comparisons between patients with and without rebleeding were performed using the Mann–Whitney U test for continuous variables and the chi‐square or Fisher's exact tests for categorical variables, as appropriate.

Multivariate Cox proportional hazards analysis was performed to identify the independent predictors of rebleeding. The proportional hazard assumption was assessed graphically and was considered acceptable. Variables considered clinically relevant were included in the multivariate model, including age, liver cirrhosis, warfarin use, aspirin use, OGIB type (overt or occult), and examination modality.

Based on the regression coefficients derived from the multivariable Cox proportional hazards model, we developed a rebleeding risk stratification score (*GIFU* score: gastrointestinal bleeding risk score in patients with fully evaluated unknown‐source bleeding). Each predictor was assigned an integer score proportional to its β coefficient.

The discriminative performance of the score was evaluated using receiver operating characteristic (ROC) curve analysis, and the area under the curve was calculated. Rebleeding rates were also evaluated according to each *GIFU* score category. For clinical applicability, patients were subsequently stratified into two risk categories according to their total score: low‐ risk (0–1 point) and high‐risk (2–3 points). In addition, cumulative rebleeding rates were estimated using the Kaplan–Meier method and compared across risk groups using the log‐rank test. Statistical significance was defined as a two‐sided *p*‐value of < 0.05. All statistical analyses were performed using JMP software (SAS Institute Inc., Cary, NC, USA).

## 3 Results

### 3.1 Patient Characteristics

A total of 226 patients diagnosed with OGIB, according to the revised Japanese definition, were included in the final analysis. The median age was 66 years (range, 11–91 years), and 145 patients (64.2%) were men. Overt OGIB was observed in 87 patients (38.4%), while 139 patients (61.6%) presented with occult OGIB. CE alone was performed in 183 patients (80.9%), and combined CE and BAE were performed in 43 patients (19.1%). The baseline characteristics of the study population are summarized in Table 1.

**TABLE 1:** Baseline characteristics of the study population.

	Overall
Total	226
Age, median (IQR), years	66 (11–91)
**Sex, *n* (%)**	
Male	145 (64.2)
Female	81 (35.8)
**OGIB type, *n* (%)**	
Overt	87 (38.4)
Occult	139 (61.5)
**Examination type, *n* (%)**	
CE	183 (80.9)
CE and BAE	43 (19.0)
**Underlying disease, *n* (%)**	
Cerebrovascular disease	15 (6.6)
Liver cirrhosis	26 (11.5)
Dialysis	12 (5.3)
Cardiovascular disease	29 (12.8)
Heart valve disease	7 (3.1)
Arrhythmia	15 (6.6)
Malignant tumor	35 (15.5)
Diabetes	35 (15.6)
**Medication, *n* (%)**	
Non‐aspirin NSAID use	22 (9.7)
Aspirin	31 (13.7)
Antiplatelet agent	49 (21.7)
Anticoagulants	21 (9.3)
Iron supplement	56 (24.8)
PPI/H2RA	105 (46.4)
Serum albumin, g/dL	3.74 ± 0.69
Hemoglobin, g/dL	9.22 ± 2.76

Abbreviations: BAE, balloon‐assisted endoscopy; CE, capsule endoscopy; H2RA, histamine H2 receptor antagonist; IQR, interquartile range; NSAID, nonsteroidal anti‐inflammatory drug; OGIB, obscure gastrointestinal bleeding; PPI, proton pump inhibitor.

### 3.2 Incidence of Rebleeding

The median time to rebleeding among patients who experienced rebleeding was 6.7 months. During the follow‐up, rebleeding occurred in 30 patients (13.2%). Patients with rebleeding underwent repeated endoscopic and/or radiological evaluations. Repeat evaluations identified the source of bleeding in seven patients. Among the seven patients who underwent repeat evaluation, a bleeding source was identified in two patients, consisting of a proximal jejunal neoplastic lesion and ileal lymphoma. In one patient, the bleeding source remained unidentified despite repeated transcatheter arterial embolization, and the patient died of bleeding‐related complications. No bleeding source was identified in the remaining four patients despite repeat evaluation.

### 3.3 Comparison Between Rebleeding and Non‐Rebleeding Groups

Clinical characteristics were compared between patients with and without rebleeding (Table 2 and Table S1). Patients who experienced rebleeding more frequently presented with overt bleeding at initial presentation (*p* < 0.01). Patients with rebleeding were more likely to undergo BAE in addition to CE (*p* < 0.01).

**TABLE 2:** Comparison of clinical characteristics between patients with and without rebleeding.

Total	Rebleeding 30	Non‐rebleeding 196	*p*‐Value
Age, median (IQR), years	70 (11–81)	66 (17–91)	0.69
**Sex, *n* (%)**			0.62
Men	20 (66.7)	125 (63.8)	
Women	10 (33.3)	71 (36.2)	
**OGIB type, *n* (%)**			< 0.01
Overt	20 (66.7)	67 (34.2)	
Occult	10 (33.3)	129 (65.8)	
**Examination type, *n* (%)**			< 0.01
CE	16 (53.3)	167 (85.2)	
CE and BAE	14 (46.7)	29 (14.8)	
**Underlying disease, *n* (%)**			
Cerebrovascular disease	3 (10)	12 (6.12)	0.42
Liver cirrhosis	9 (30)	17 (8.6)	< 0.01
Dialysis	2 (6.7)	10 (5.1)	0.66
Cardiovascular disease	4 (13.3)	25 (12.7)	1.00
Heart valve disease	1 (3.3)	6 (3.1)	1.00
Arrhythmia	1 (3.3)	14 (7.1)	0.69
Malignant tumor	4 (13.3)	31 (15.8)	1.00
Diabetes	6 (20.0)	29 (14.8)	0.42
**Medication, *n* (%)**			
Non‐aspirin NSAID use	2 (6.7)	20 (10.2)	0.74
Aspirin	14 (26.9)	17 (9.8)	< 0.01
Antiplatelet agent	11 (36.7)	38 (19.5)	0.05
Anticoagulants	4 (13.3)	17 (8.7)	0.49
Iron supplement	8 (26.7)	48 (24.5)	0.82
PPI/H2RA	15 (50.0)	90 (45.9)	0.69
Serum albumin, median ± SD, g/dL	3.77 ± 0.55	3.76 ± 0.65	0.92
Hemoglobin, median ± SD, g/dL	8.93 ± 2.11	9.32 ± 2.78	0.45
Median follow‐up duration, months (IQR)	20.5 (8.6–48.7)	4.6(0.6–36.2)	0.21

Abbreviations: BAE, balloon‐assisted endoscopy; CE, capsule endoscopy; H2RA, histamine H2 receptor antagonist; NSAIDs, nonsteroidal anti‐inflammatory drugs; OGIB, obscure gastrointestinal bleeding; PPI, proton pump inhibitor; SD, standard deviation.

Regarding comorbidities and medication use, liver cirrhosis (*p* < 0.01) and aspirin (*p* < 0.01) were significantly more common in the rebleeding group. In contrast, we found no significant differences between patients with and without rebleeding with respect to age, sex, or the use of other antiplatelet agents or anticoagulants.

### 3.4 Predictors of Rebleeding

In multivariable Cox proportional hazards analysis, liver cirrhosis, aspirin use, and overt OGIB were identified as independent predictors of rebleeding. Age and examination modality were not significantly associated with rebleeding after adjustment (Table 3). An additional multivariable analysis, including non‐aspirin NSAID use, was performed. Non‐aspirin NSAID use was not independently associated with rebleeding, whereas aspirin use remained significant (Table S2).

**TABLE 3:** Multivariable Cox proportional hazards analysis for predictors of rebleeding.

Variable		HR	95% CI	*p*‐Value	β	Assigned points
Liver cirrhosis	Yes	3.81	1.44–10.2	<0.01	0.59	1
	No	1				
Examination modality	CE	1.45	0.50–4.21	0.48		
	CE+BAE	1				
Warfarin use	Yes	3.12	0.80–12.1	0.09		
	No	1				
Age	≧71	1.53	0.73–3.21	0.25		
	<71	1				
Aspirin use	Present	2.83	1.14–6.97	0.02	0.51	1
	Absent	1				
OGIB type	Overt	4.06	1.52–11.1	< 0.01	0.69	1
	Occult	1				

Abbreviations: BAE, balloon‐assisted endoscopy; CE, capsule endoscopy; CI, confidence interval; HR, hazard ratio; OGIB, obscure gastrointestinal bleeding.

### 3.5 Development of the *GIFU* Score

Based on the regression coefficients derived from the multivariable Cox proportional hazards model, the rebleeding risk stratification score, the *GIFU* score (gastrointestinal bleeding risk scores were developed for patients with fully evaluated unknown‐source bleeding), was developed. Each independent predictor was assigned an integer score proportional to its β coefficient (Table 3).

Each independent predictor was assigned one point, yielding a total *GIFU* score ranging from 0 to 3 points.

### 3.6 Discriminative Performance of the Risk Score

**FIGURE 2:**
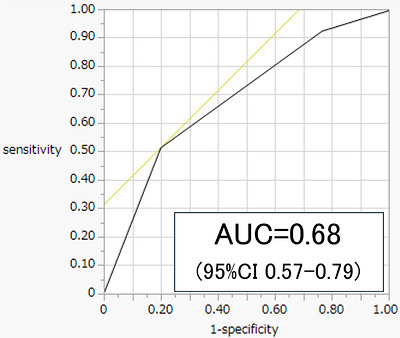
Receiver operating characteristic (ROC) curve of the *GIFU* score for predicting rebleeding. The ROC curve demonstrated the discriminative performance of the rebleeding risk (*GIFU)* score for predicting rebleeding in patients with obscure gastrointestinal bleeding (OGIB) according to the revised Japanese definition. The area under the curve (AUC) was 0.68 (95%CI, 0.57–0.79), indicating modest discrimination. Using a cut‐off value of ≥2 points, the sensitivity and specificity were 23.3% and 91.8%, respectively.

The *GIFU* score demonstrated a modest discriminative ability to predict rebleeding (Figure 2). ROC curve analysis yielded an area under the curve of 0.68 (95% confidence interval, 0.57–0.79), indicating modest discrimination for rebleeding prediction. Using a cut‐off value of ≥2 points, the sensitivity and specificity for predicting rebleeding were 23.3% and 91.8%, respectively.

The overall rebleeding rates increased progressively with increasing *GIFU* scores: 5.6% in patients with a score of 0, 17.5% in those with a score of 1, 27.3% in those with a score of 2, and 100% in those with a score of 3. Kaplan–Meier analysis stratified by the four *GIFU* score categories also demonstrated a stepwise increase in cumulative rebleeding risk (Figure S1).

### 3.7 Cumulative Incidence of Rebleeding According to Risk Categories

**FIGURE 3:**
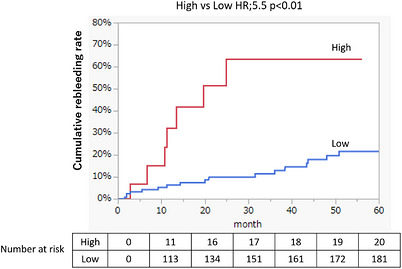
Kaplan–Meier analysis of cumulative rebleeding incidence stratified by *GIFU* risk categories. Patients were stratified into low‐risk (0–1 point) and high‐risk (2–3 points) groups according to their *GIFU* scores. The cumulative incidence of rebleeding was significantly higher in the high‐risk group than in the low‐risk group (log‐rank test, *p* < 0.01).

Kaplan–Meier analysis revealed a significantly higher cumulative incidence of rebleeding in the high‐risk group than in the low‐risk group (log‐rank test, *p* < 0.01) (Figure 3). The low‐risk group showed consistently low rebleeding rates throughout the follow‐up period.

## 4 Discussion

In this study, rebleeding remained clinically relevant in patients with OGIB despite a comprehensive gastrointestinal evaluation with negative findings. Liver cirrhosis, aspirin use, and overt OGIB were identified as independent predictors of rebleeding, and a simple risk‐stratification model demonstrated modest utility in identifying high‐risk patients.

To the best of our knowledge, this is one of the few studies specifically investigating rebleeding risk in patients who underwent comprehensive evaluation of the upper, lower, and small bowels without the identification of a definite bleeding source. Although several studies have reported outcomes in patients with negative findings on CE [10, 14, 15, 16, 17, 18, 19, 20], the definition of negative findings varies considerably. In some reports, lesions corresponding to the P0 or P1 categories of the Saurin classification [21] were included [15, 16, 17, 18], whereas in others, the criteria for negative findings were not explicitly stated [15, 19, 20]. By restricting the analysis to patients with negative findings after comprehensive gastrointestinal evaluation, our study evaluated the rebleeding risk in a more clearly defined population.

Several risk‐stratification models and clinical scoring systems have been proposed to predict rebleeding in patients with small‐bowel OGIB [22, 23, 24], and a number of risk factors, including age [14, 24], sex [9], overt OGIB [9, 25, 26], anticoagulant use [9, 15, 26], and chronic renal failure [24, 27, 28], have been reported across different cohorts. We have previously demonstrated that liver cirrhosis is a significant risk factor for rebleeding in patients with OGIB [29]. Although many existing risk scores were developed in heterogeneous populations that included patients with identifiable small‐bowel lesions or variably defined negative findings, liver cirrhosis [9, 26, 29], aspirin use [24, 30], and overt OGIB [9, 25, 26] were identified as independent predictors of rebleeding in the present study, consistent with previous reports. These factors may collectively reflect underlying vulnerabilities, including impaired hemostasis associated with liver cirrhosis, aspirin‐related mucosal injury, and clinically aggressive bleeding phenotype in patients with overt OGIB. In patients with liver cirrhosis, progressive portal hypertension may exacerbate small‐bowel abnormalities and increase susceptibility to recurrent bleeding [30]. Although these predictors are not entirely novel, the present study provides a practical risk‐stratification approach, specifically for patients with OGIB after a comprehensive gastrointestinal evaluation with negative findings, a population that has been insufficiently characterized in previous studies.

Another important finding of this study was that examination modality was not independently associated with rebleeding after adjustment. Although missed or intermittent bleeding lesions cannot be completely excluded, particularly in the proximal small bowel, where CE visualization may be limited [32, 33], previous studies have demonstrated that CE has a high diagnostic performance for small‐bowel pathology [34]. Nevertheless, repeat evaluations identified bleeding sources in only a subset of patients during follow‐up. Furthermore, one patient died of hemorrhagic shock despite an initially negative comprehensive gastrointestinal evaluation, highlighting that clinically significant rebleeding may still occur in this population. These findings suggest that recurrent bleeding cannot be explained solely by differences in diagnostic strategies or missed lesions. Rather, baseline clinical vulnerability appeared to play a central role in determining the risk of rebleeding. Therefore, risk stratification may be important for identifying patients at higher risk of rebleeding after an initial negative comprehensive gastrointestinal evaluation.

The proposed *GIFU* score demonstrated a modest discriminative ability for predicting rebleeding. Although its predictive performance was moderate, the score was based on simple clinical variables that are readily available in routine practice and may, therefore, provide practical value for risk stratification. In particular, patients classified as high‐risk showed significantly higher cumulative rebleeding rates than those classified as low‐risk. These findings support the clinical utility of the GIFU score for risk stratification after a negative comprehensive gastrointestinal evaluation.

This study had several limitations. First, this was a retrospective, single‐center study, which may have limited the generalizability of our findings. Second, despite a comprehensive gastrointestinal evaluation, the possibility of missed or intermittent bleeding lesions cannot be completely excluded. Third, follow‐up information was obtained from our institutional medical records. Because some patients continued follow‐up at referring hospitals or community clinics after the initial evaluation, subsequent rebleeding events may not have been fully captured. Therefore, the true incidence of rebleeding may have been underestimated, which could have affected the precision of the risk estimates and the proposed risk model. Fourth, the *GIFU* score was derived and evaluated within the same single‐center cohort. Although we explored internal validation approaches, the limited number of rebleeding events precluded reliable model validation. Therefore, overfitting cannot be excluded, and prospective external validation in independent cohorts is required before routine clinical application.

In conclusion, rebleeding remained clinically relevant in patients with OGIB despite a comprehensive gastrointestinal evaluation with negative findings. Importantly, patients with OGIB were not a homogeneous population, and the risk of rebleeding varied substantially according to baseline clinical characteristics. The *GIFU* score, based on liver cirrhosis, overt OGIB, and aspirin use, provides a simple and practical tool for identifying patients at increased risk of rebleeding after a negative comprehensive gastrointestinal evaluation. By distinguishing patients with different risks of rebleeding, the *GIFU* score may support risk‐adapted follow‐up strategies and facilitate more efficient allocation of specialist care. Our findings suggest that rebleeding in OGIB reflects baseline clinical vulnerability more strongly than differences in examination modality. Future prospective studies are needed to determine the optimal follow‐up strategy according to risk category and to further validate the clinical utility of the *GIFU* score.

## Author Contributions

**Sachiyo Onishi** wrote the manuscript. **Sachiyo Onishi**, **Kiichi Otani**, **Naoya Masuda**, **Hiroki Taniguchi**, **Kentaro Kojima**, **Jun Takada**, **Masaya Kubota**, **Takashi Ibuka**, and **Masahito Shimizu** managed the patients. All authors discussed the results and commented on the manuscript.

## Funding

No funding was received for this study. The authors have nothing to report.

## Ethics Statement

The study protocol was reviewed and approved by the Institutional Review Board of Gifu University Hospital.

## Consent

The opt‐out method was used to obtain consent from the study participants.

## Conflicts of Interest

The authors declare no conflicts of interest. The authors have nothing to report.

## Supporting information




**Supporting File**: deo270378‐sup‐0001‐SuppMat.docx

## Data Availability

The data that support the findings of this study are available from the corresponding author upon reasonable request.
