# Supplementary material for: Risk Stratification for Rebleeding in Obscure Gastrointestinal Bleeding After Comprehensive Gastrointestinal Evaluation
Source: DEN Open. 2026 Jul 6;7(1):e70378. doi: 10.1002/deo2.70378 (PMC13334218; doi:10.1002/deo2.70378)
Supplement: Supplementary file 1 — Supporting File: deo270378‐sup‐0001‐SuppMat.docx [file DEO2-7-e70378-s001.docx]

Supplementary table S1

| n | | | rebleeding | non rebleeding |
| --- | --- | --- | --- | --- |
|  |  |  | 30 | 196 |
| GIFU score | low risk | 203 | 23 | 180 |
|  | high risk | 23 | 7 | 16 |

Supplementary table S2

| Variable | | HR | 95% CI | p-value |
| --- | --- | --- | --- | --- |
| Liver cirrhosis | yes | 3.32 | 1.33-8.31 | 0.01 |
|  | no | 1 |  |  |
| Examination type | CE | 1.45 | 0.48-4.14 | 0.522 |
|  | CE+BAE | 1 |  |  |
| Medication Warfarin | yes | 3.26 | 0.82-12.8 | 0.09 |
|  | no | 1 |  |  |
| Age | ≧71 | 1.12 | 0.52-2.44 | 0.76 |
|  | <71 | 1 |  |  |
| Medication Aspirin | present | 2.81 | 1.12-7.05 | 0.02 |
|  | absent | 1 |  |  |
| Non-aspirin NSAID use | present | 1.23 | 0.27-5.64 | 0.78 |
|  | absent | 1 |  |  |
| OGIB type | overt | 4.08 | 1.48-11.2 | <0.01 |
|  | occult | 1 |  |  |

HR, hazard ratio; CI, confidence interval; CE, capsule endoscopy; BAE, balloon-assisted endoscopy; NSAID, Non-Steroidal Anti-Inflammatory Drug; OGIB, obscure gastrointestinal bleeding;


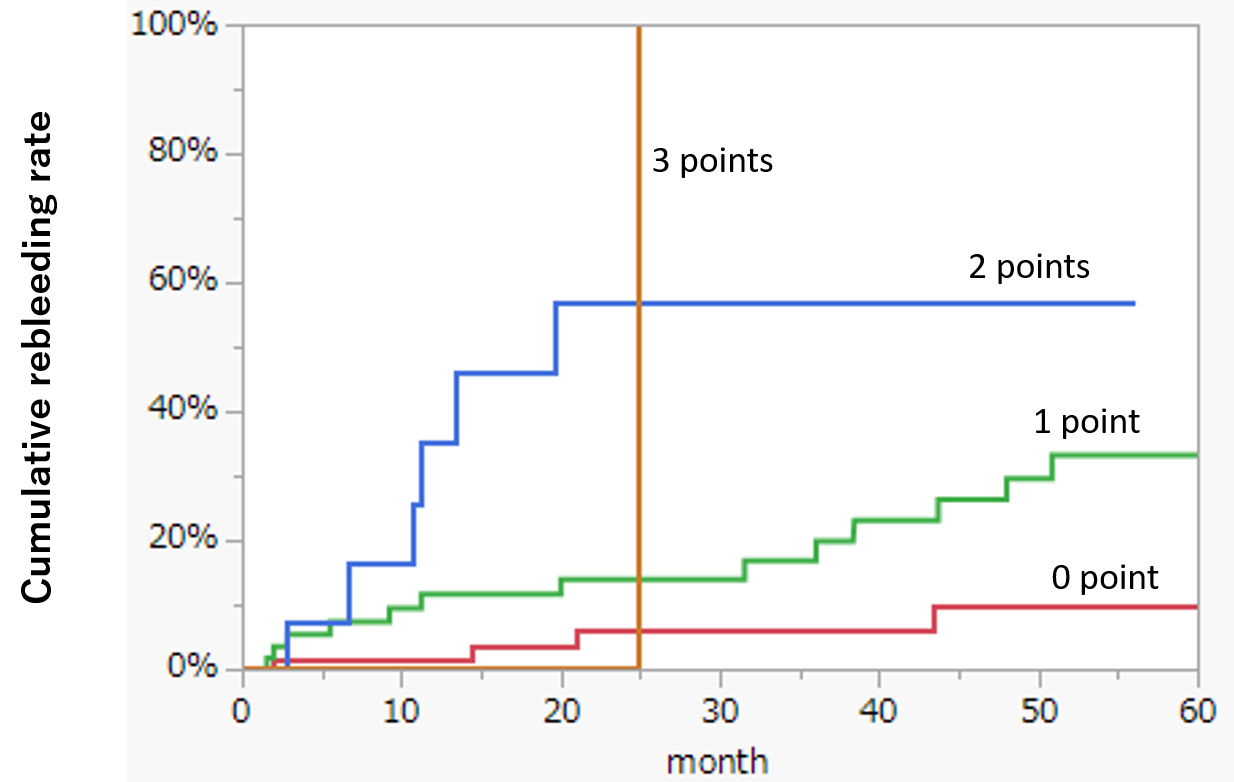
Supplementary Figure S1
